# Supplementary material for: Attention-based deep learning for analysis of pathology images and gene expression data in lung squamous premalignant lesions
Source: Genome Med. 2026 Apr 8;18:69. doi: 10.1186/s13073-026-01636-8 (PMC13188385; doi:10.1186/s13073-026-01636-8)
Supplement: Supplementary file 1 — Additional file 1: Figure S1. Whole slide image pre-processing pipeline. Figure S2. Gene expression data pre-processing pipeline. Figure S3. Data split and cross-validation scheme. Figure S4. Heatmap of statistical significance between prediction probabilities grouped by histology grade. Figure S5. Scatter plot of external testing sample prediction probabilities. Figure S6. Principal Component (PC) Analysis plots on external testing samples by cohort (extended from Fig. 2H and Fig. 3G). Figure S7. Gene heatmap of external testing biopsy samples. Table S1. Comparison of model performance with external multimodal models. Table S2. Accuracy, sensitivity, and specificity across histologic grades. [file 13073_2026_1636_MOESM1_ESM.docx]

**Figure S1. Whole slide image pre-processing pipeline.** WSIs were processed by a pipeline that separated tissues from backgrounds and artifacts, meanwhile generating squared image patches and constructing them into an undirected graph. Patch embeddings were computed using CTransPath and used as node features in the graph. Edges were built upon 8-connectivity.

**Figure S2. Gene expression data pre-processing pipeline.** All training datasets were batch corrected using PCA Dataset 1 discovery cohort as the reference dataset across the intersection set of genes. Ensembl transcript IDs were mapped to Ensembl gene IDs before intersection. Patient-level stratified sampling (see **Figure S3** for details) was conducted within each dataset, followed by sample reorganization by fold. Within each fold, a linear mixed-effects regression was implemented, and the resulting t-statistics were used to sort genes associated with dysplasia or worse histology. The topmost significant 100 up- and down- regulated genes were selected per fold. For external testing, datasets were batch corrected to PCA Dataset 1 discovery cohort and then subset to the selected sets.


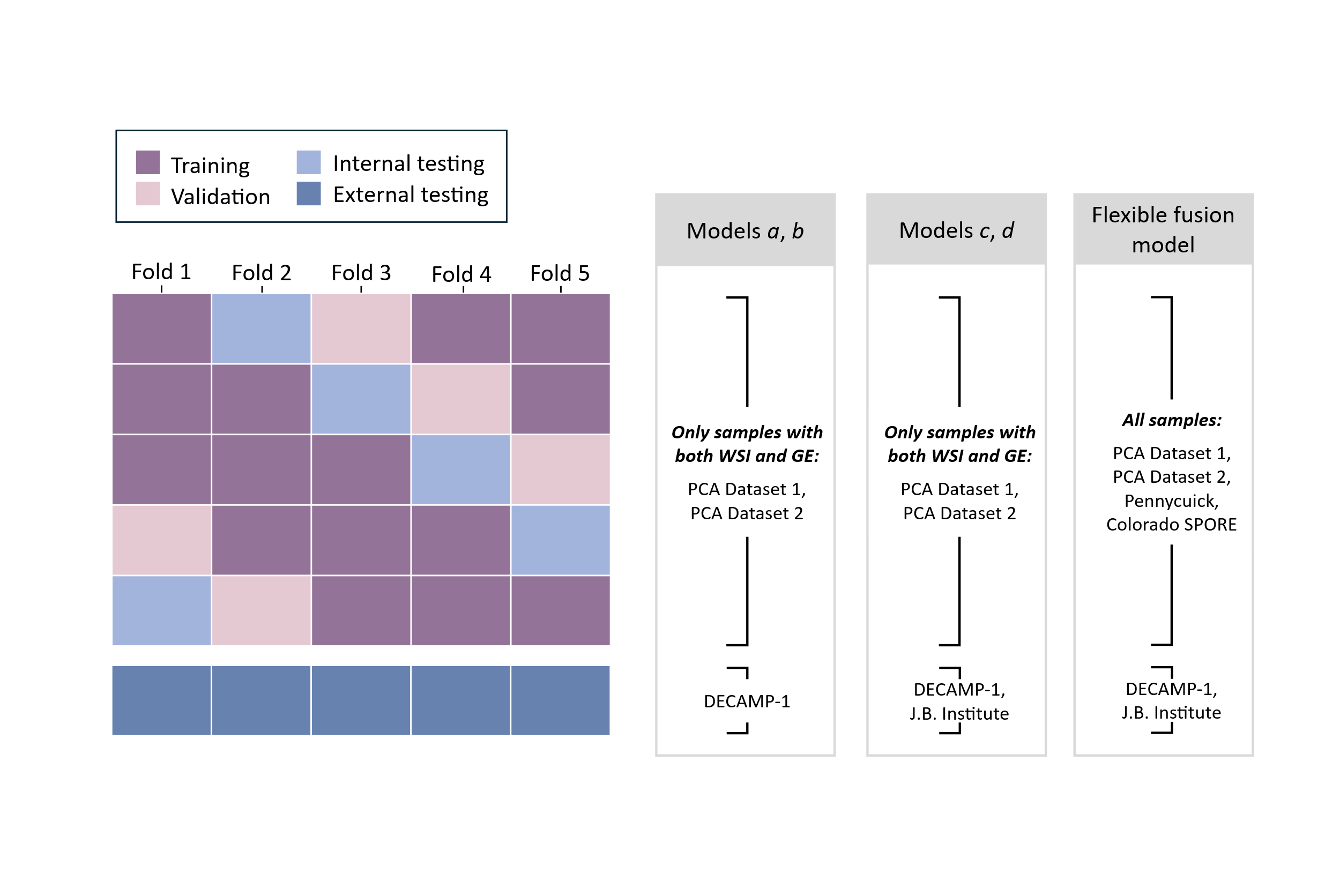


**Figure S3. Data split and cross-validation scheme.** Models were trained and internally tested on PCA Dataset 1 & 2, Pennycuick, and Colorado SPORE cohorts, using samples with WSI (Model *a*), samples with GE (Model *c*), samples with both WSI and GE (Model *b* and Model *d*), or all samples (flexible fusion model). Samples from each dataset were randomly divided into five folds through a stratified split at the patient-level and then combined by fold. Models were externally tested on DECAMP-1 and Jules Bordet Institute cohorts.


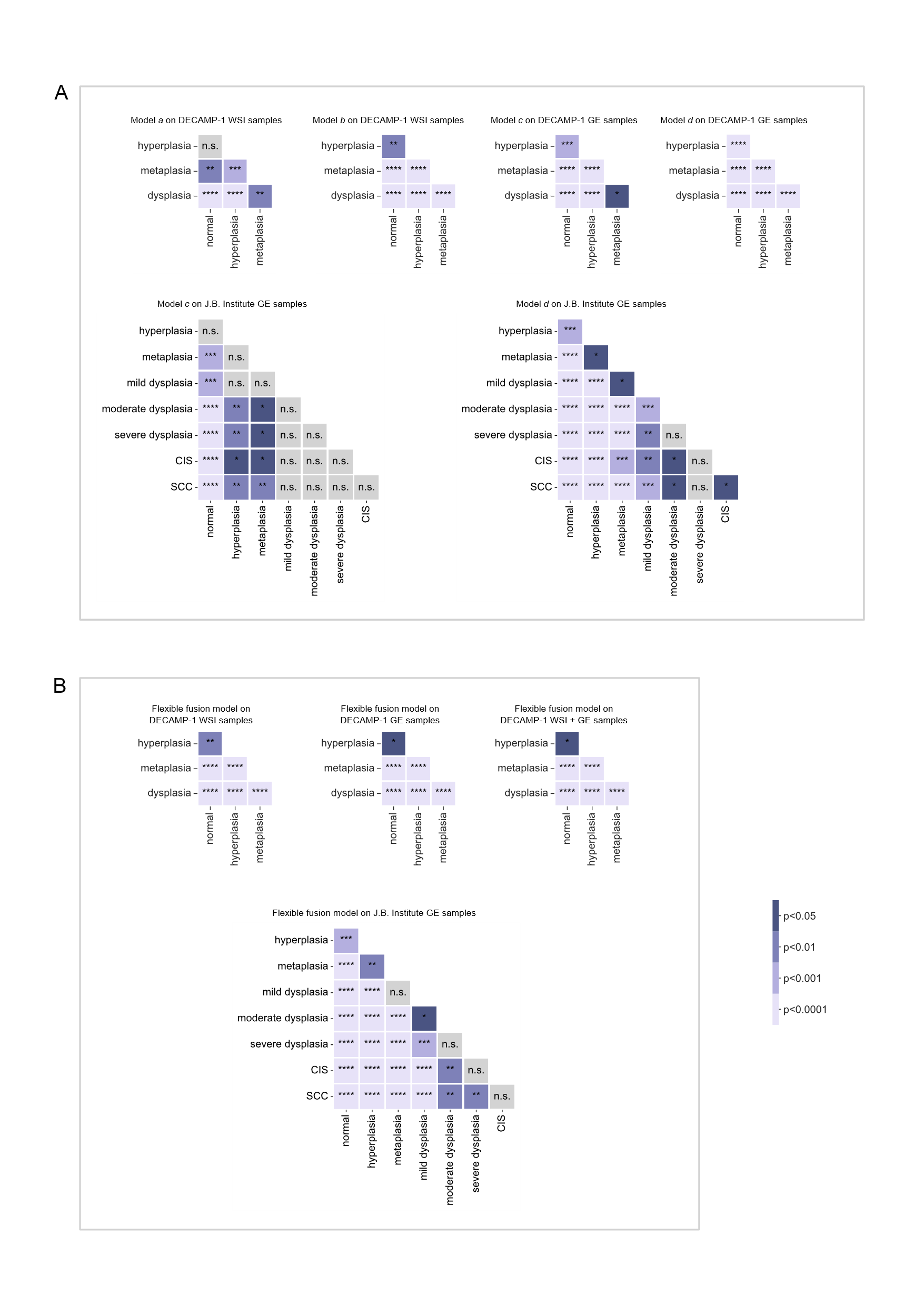


**Figure S4. Heatmap of statistical significance between prediction probabilities grouped by histology grade.** The prediction probabilities group by histology grade is represented boxplots from **Figure 2** and **Figure 3.** **A.** P-values of all histologic grade pairs in boxplots **Figures 2D-2G**. **B.** P-values of all histologic grade pairs in boxplots **Figures 3D-3F**. Significance levels are denoted as * for p<0.05; ** for p<0.01; *** for p<0.001; and **** for p<0.0001.


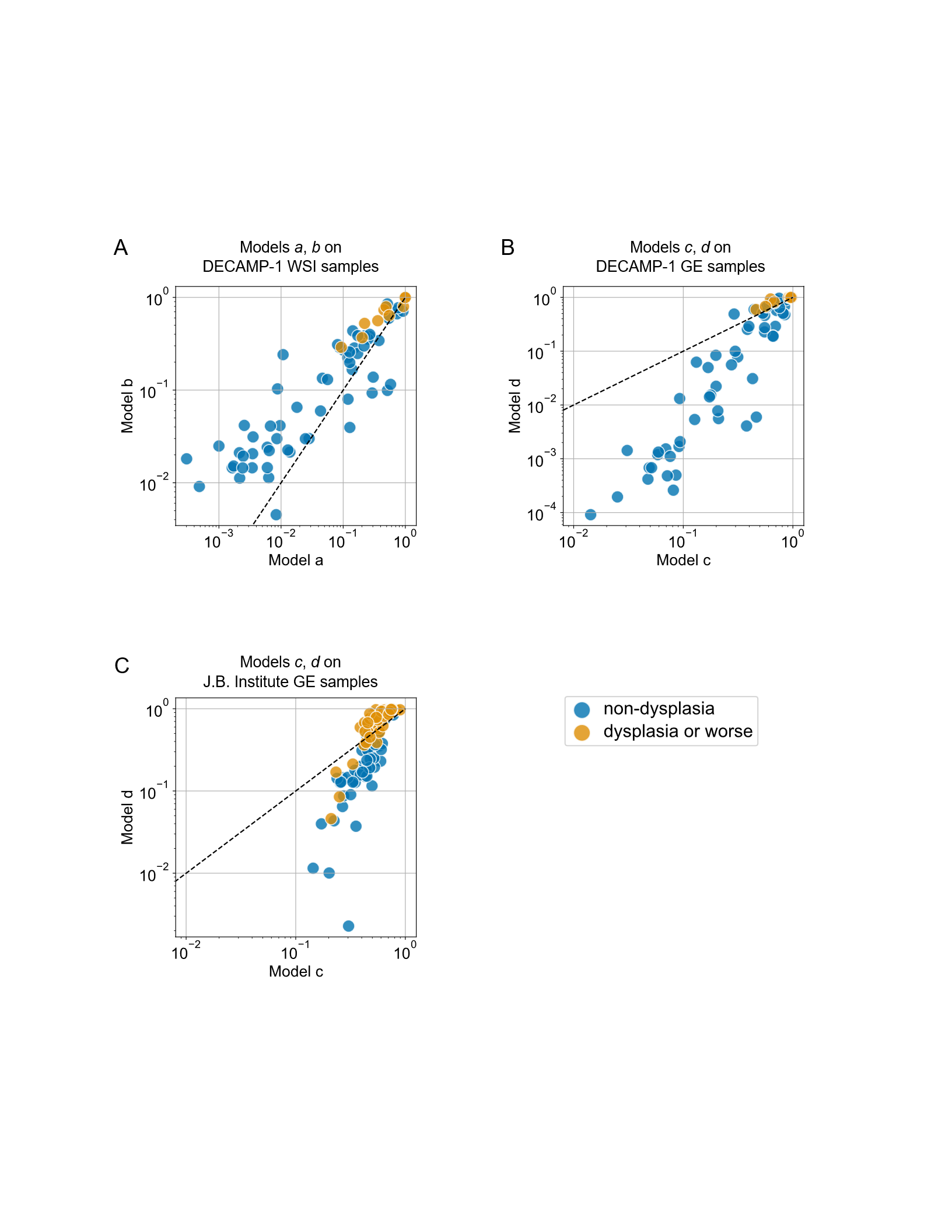


**Figure S5. Scatter plot of external testing sample prediction probabilities.** Categorized by histologic grades, sample prediction probabilities were plotted for Model *a* against Model *b* on DECAMP-1 WSI samples (**A**), Model *c* against Model *d* on DECAMP-1 GE samples (**B**), and Model *c* against Model *d* on J.B. Institute GE samples (**C**).


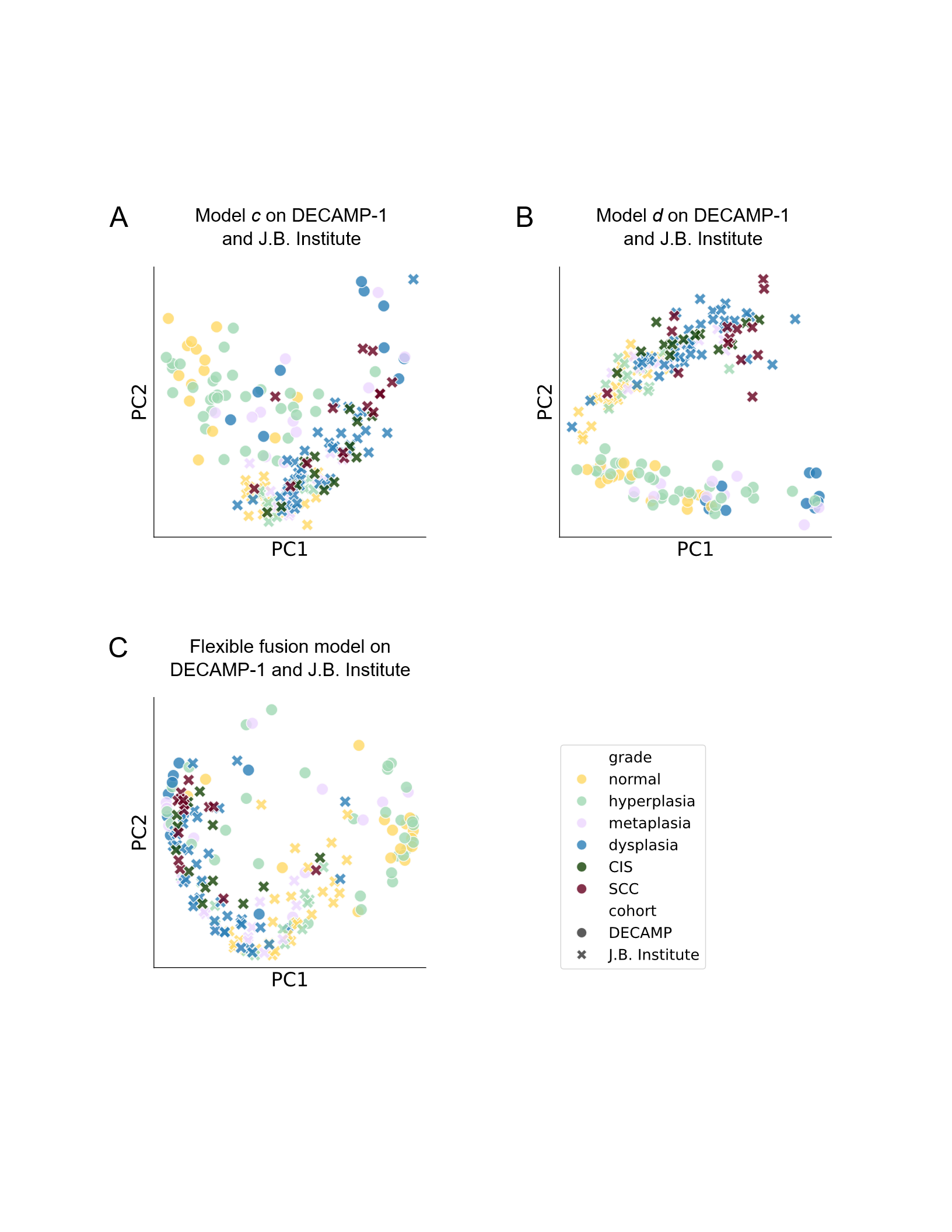


**Figure S6. Principal Component (PC) Analysis plots on external testing samples by cohort (extended from Figure 2H and Figure 3G).** PC1 (x-axis) is plotted versus PC2 (y-axis)**. A.** Model *c* on DECAMP-1 and J.B. Institute GE samples. **B**. Model *d* on DECAMP-1 and J.B. Institute GE samples. **C**. The flexible fusion model on DECAMP-1 and J.B. Institute GE samples.


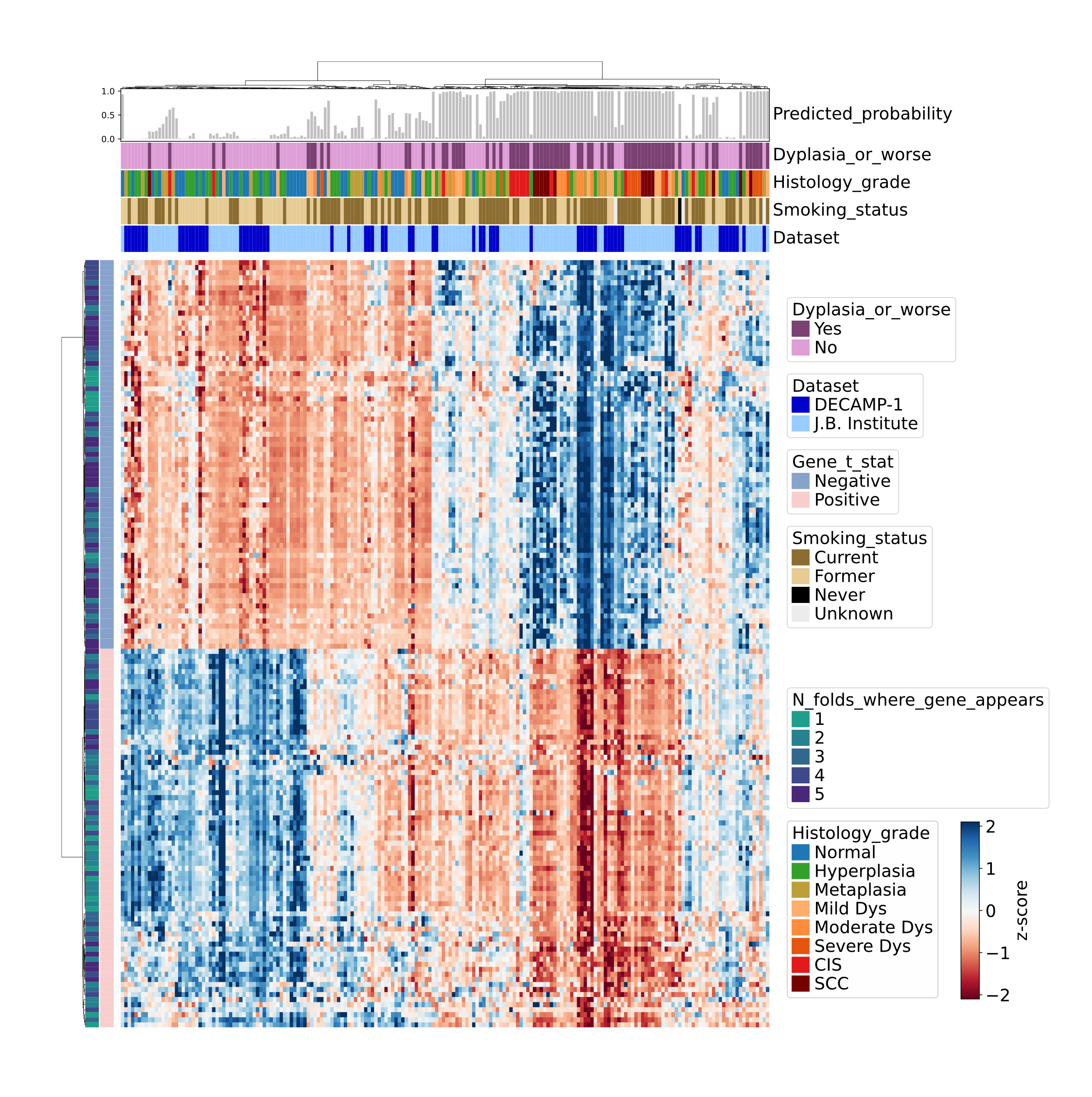


**Figure S7**. **Gene heatmap of external testing biopsy samples.** Gene expression values (z-score normalized log cpm for RNA-seq and log RMA values for microarray) of the external testing samples subset to the selected top genes from the best-performing fold of the flexible fusion model. Selected top genes absent from external testing samples were excluded (one from DECAMP and 47 from J.B. Institute). Top annotations indicate predicted probabilities, sample labels (dysplasia or not), histology grades (normal, hyperplasia, metaplasia, mild dysplasia, moderate dysplasia, severe dysplasia, CIS, or SCC), smoking status (current, former, never, or unknown), and cohort information (DECAMP-1 or J.B. Institute). Left annotations show the number of folds where the gene appears (1 to 5) and the sign of the t-statistic for the histology grade based on the linear regression using training data. Rows and columns were clustered using the Ward method.

|  | Internal testing | | | | |
| --- | --- | --- | --- | --- | --- |
|  | AUROC | Accuracy | Precision | Recall | Specificity |
| PathomicFusion (CNN ⊗ SNN) | 0.864±0.043 | 0.852±0.020 | 0.849±0.071 | 0.834±0.063 | 0.829±0.081 |
| PORPOISE | 0.878±0.028 | 0.875±0.031 | 0.865±0.053 | 0.838±0.042 | 0.876±0.035 |
| Model *b* (*d*) (ours) | 0.877±0.016 | 0.844±0.055 | 0.837±0.052 | 0.821±0.076 | 0.851±0.061 |
| Flexible fusion model (ours) | 0.957±0.017 | 0.883±0.060 | 0.865±0.037 | 0.873±0.149 | 0.902±0.044 |

|  | External testing | | | | |
| --- | --- | --- | --- | --- | --- |
|  | AUROC | Accuracy | Precision | Recall | Specificity |
| PathomicFusion (CNN ⊗ SNN) | 0.835±0.043 | 0.801±0.056 | 0.632±0.039 | 0.728±0.061 | 0.816±0.037 |
| PORPOISE | 0.827±0.062 | 0.792±0.038 | 0.571±0.072 | 0.673±0.052 | 0.787±0.019 |
| Model *b* (*d*) (ours) | 0.898±0.036 | 0.854±0.023 | 0.487±0.068 | 0.645±0.072 | 0.892±0.026 |
| Flexible fusion model (ours) | 0.954±0.009 | 0.849±0.058 | 0.508±0.096 | 0.960±0.049 | 0.830±0.073 |

**Table S1. Comparison of model performance with external multimodal models.** We compared our models (Model *b* (*d*) and the flexible fusion model) with two external pathology-gene fusion models, PathomicFusion (CNN ⊗ SNN) and PORPOISE. All models were trained, validated, and internally and externally tested using the 5-fold cross-validation experiment setting detailed in Figure S3. PathomicFusion, PORPOISE, and Model *b* (*d*) were trained and tested on samples with both WSI and GE, whereas the flexible fusion model was trained on samples with WSI or GE or both and tested on samples with both WSI and GE. Internal testing was performed on 328 samples from PCA Dataset1 and Dataset 2. External testing was performed on 70 samples with from DECAMP-1 cohort. Mean and standard deviation of AUROC, accuracy, precision, recall, and specificity across were reported for each model.

|  | Accuracy | | Sensitivity | | Specificity | |  |
| --- | --- | --- | --- | --- | --- | --- | --- |
| Model *a* | | | | | | |  |
| normal | 0.893±0.033 | | N/A | | 0.893±0.033 | |  |
| hyperplasia | 0.909±0.033 | | N/A | | 0.909±0.033 | |  |
| metaplasia | 0.783±0.085 | | N/A | | 0.783±0.085 | |  |
| dysplasia | 0.480±0.147 | | 0.480±0.147 | | N/A | |  |
| Model *b* | | | | | | |  |
| normal | 0.987±0.027 | | N/A | | 0.987±0.027 | |  |
| hyperplasia | 0.891±0.053 | | N/A | | 0.891±0.053 | |  |
| metaplasia | 0.650±0.082 | | N/A | | 0.650±0.082 | |  |
| dysplasia | 0.640±0.196 | | 0.640±0.196 | | N/A | |  |
| Model *c* | | | | | | |  |
| normal | 0.752±0.288 | | N/A | | 0.752±0.288 | |  |
| hyperplasia | 0.700±0.238 | | N/A | | 0.700±0.238 | |  |
| metaplasia | 0.496±0.340 | | N/A | | 0.496±0.340 | |  |
| dysplasia | 0.542±0.290 | | 0.542±0.290 | | N/A | |  |
| CIS | 0.431±0.403 | | 0.431±0.403 | | N/A | |  |
| SCC | 0.471±0.380 | | 0.471±0.380 | | N/A | |  |
| Model *d* | | | | | | |  |
| normal | 0.924±0.152 | | N/A | | 0.924±0.152 | |  |
| hyperplasia | 0.829±0.143 | | N/A | | 0.829±0.143 | |  |
| metaplasia | 0.556±0.274 | | N/A | | 0.556±0.274 | |  |
| dysplasia | 0.658±0.179 | | 0.658±0.179 | | N/A | |  |
| CIS | 0.523±0.235 | | 0.523±0.235 | | N/A | |  |
| SCC | 0.729±0.114 | | 0.729±0.114 | | N/A | |  |
| Flexible fusion model (tested on WSI) | | | | | | | |
| normal | | 0.867±0.140 | | N/A | | 0.867±0.140 | |
| hyperplasia | | 0.812±0.212 | | N/A | | 0.812±0.212 | |
| metaplasia | | 0.567±0.286 | | N/A | | 0.567±0.286 | |
| dysplasia | | 0.840±0.049 | | 0.840±0.049 | | N/A | |
| Flexible fusion model (tested on GE) | | | | | | | |
| normal | | 0.886±0.112 | | N/A | | 0.886±0.112 | |
| hyperplasia | | 0.850±0.099 | | N/A | | 0.850±0.099 | |
| metaplasia | | 0.504±0.147 | | N/A | | 0.504±0.147 | |
| dysplasia | | 0.754±0.135 | | 0.754±0.135 | | N/A | |
| CIS | | 0.862±0.132 | | 0.862±0.132 | | N/A | |
| SCC | | 0.914±0.070 | | 0.914±0.070 | | N/A | |
| Flexible fusion model (tested on WSI+GE) | | | | | | | |
| normal | | 0.947±0.050 | | N/A | | 0.947±0.050 | |
| hyperplasia | | 0.897±0.045 | | N/A | | 0.897±0.045 | |
| metaplasia | | 0.500±0.321 | | N/A | | 0.500±0.321 | |
| dysplasia | | 0.960±0.049 | | 0.960±0.049 | | N/A | |

**Table S2. Accuracy, sensitivity, and specificity across histologic grades.** Model performance on the external testing set within each histologic grade (normal, hyperplasia, metaplasia, dysplasia, CIS, or SCC), expressed as mean ± SD across five cross-validation folds. The number of histologic grades depends on the input datasets. Per-grade accuracy, sensitivity, and specificity were derived from confusion matrices computed separately for each fold.
